# Quality perception, purchase intention, and the impact of information on the evaluation of refined Pacific cupped oysters (Crassostrea gigas) by Dutch consumers

**DOI:** 10.1002/jsfa.9136

**Published:** 2018-06-26

**Authors:** Jasper van Houcke, Themistokilis Altintzoglou, Jozef Linssen, Joop Luten

**Affiliations:** ^1^ Department of Delta Academy, HZ University of Applied Sciences Vlissingen The Netherlands; ^2^ Department of Agrotechnology and Food Sciences, Food Quality and Design Wageningen University Wageningen The Netherlands; ^3^ Department of Consumer and Marketing Research Nofima Tromsø Norway

**Keywords:** Crassostrea gigas, refinement, purchase intention, quality, product evaluation, consumer

## Abstract

**BACKGROUND:**

Oyster refinement using land‐based pond systems is a new activity in the Dutch oyster sector. It increases the oyster's tissue weight and changes its sensorial properties. However, the response of Dutch consumers towards refined oysters is unknown. The research aim was to gain insight into the importance of oyster quality parameters, drivers for oyster consumption, and acceptance of refined oysters by Dutch consumers, taking into account the information given to them about the product and process.

**RESULTS:**

Taste, texture, and odor are the most important oyster quality characteristics for Dutch consumers. The outcome of questionnaires showed that willingness to buy and pay is influenced by factors such as the oysters' country of origin, cultivation area, and flavor profile. Refinement did not affect willingness to buy and pay. Furthermore Dutch consumers seem to have a preference for the flavor profile of refined oysters. Consumer evaluation showed that refined Pacific cupped oysters were perceived as sweeter compared with non‐refined oysters. When information on the cultivation process was disclosed, overall appreciation of refined oysters by consumers increased.

**CONCLUSION:**

New insights in the importance of oyster quality characteristics for Dutch consumers are generated that can be used in the development of refined Pacific cupped oysters. © 2018 The Authors. *Journal of The Science of Food and Agriculture* published by John Wiley & Sons Ltd on behalf of Society of Chemical Industry.

## INTRODUCTION

Oyster refinement, or fattening of oysters using land‐based pond systems, is a new activity for the oyster sector in the Netherlands. In oyster refinement, market‐sized Pacific cupped oysters (*Crassostrea gigas*, Thunberg) are kept in basins and fed with algae to increase the oyster tissue weight and to change the sensorial properties of the oysters.^1^, ^2^ Differences in odor, taste, and appearance between refined and non‐refined oysters have been shown when trained panelists were used for the evaluation.^3^, ^4^ Refined Pacific cupped oysters have been characterized by a stronger grass odor, a sweeter, less salty and less bitter flavor, in comparison to non‐refined oysters.^3^ Furthermore refined Pacific cupped oysters have also been reported to have a lower overall odor intensity and marine flavor.^4^ In appearance, the tissue of the refined oysters seemed to be larger in comparison with non‐refined oysters and the color of the visceral mass seems to be whiter. Furthermore, it was shown that naïve consumers were able to discriminate between refined and non‐refined oysters in sensory evaluations.^4^

The profile of Dutch oyster consumers could be described as predominantly male, over 55 years of age with a relatively high educational level and gross yearly household income.^4^, ^5^ This consumer profile is very similar to that found in a French study.^6^ Oyster consumers could be regarded as traditionalists in their choices and preferences regarding oyster products. Debucquet^6^ showed that acceptance of new oyster products was influenced by the age of the consumers and whether the consumers were eating oysters on a regular basis. Their study evaluated different products containing oysters as an ingredient and with different processing levels. The products used in that study were cooked oysters in a half shell, hot preparation for toast, potted oyster, oyster butter, and oyster‐based soup. When it was mentioned that the evaluated products included oysters as one of the ingredients, the opinions of the participating consumers changed towards a more negative evaluation. The authors^6^ attributed this change to disgust from non‐regular oyster consumers. On the other hand, the changes in evaluations by regular oyster consumers were attributed to a loss of the product's naturalness or downgrading of a delicacy. These findings stress the importance of understanding consumers' attitudes before entering the market with new oyster products.

In many cases new products are launched into the market based upon intrinsic (e.g., appearance, taste, odor, texture) and extrinsic product characteristics (e.g., brand, packaging, nutritional and health claims). However, consumer food choice is more complex than that. Other factors like biological, psychological, situational, and socio‐cultural factors also play a role in consumer food choices.^7^ In particular, in the case of refined oysters, which could be considered as a new product, neophobia, trust in food technology, and other cultural and economical influences might play a role in consumer acceptance. The acceptance of a new food technology, like refinement, depends on the perceived benefits, risks, and naturalness of the process and product. Information about the benefits of the new technology and consumer trust has been reported as being essential for consumer acceptance.^8^ Product characteristics seem less important to consumers of luxury products as consumer satisfaction comes from the response of other people to the display of wealth and status of the luxury products.^9^, ^10^

Refined oysters are nowadays sold on the Dutch market as luxury products for a premium price. However little is known about the purchase drivers and acceptance of new oyster products by Dutch consumers. The aim of this study was to gain insight into the importance of oyster quality parameters, drivers for oyster consumption, and acceptance of refined oysters by Dutch consumers.

## MATERIALS AND METHODS

Three independent studies with Dutch consumers were performed. In the first study the importance of a number of parameters relevant for the perception of the quality of oysters by Dutch consumers was evaluated using questionnaires. In the second study questionnaires were used to evaluate the effects of the information about the cultivation process (refinement versus no refinement of oysters) and other purchase intention drivers such as country of origin, cultivation area, and flavor profile on Dutch consumers' willingness to buy and to pay for new oyster products. In the third study, actual products, refined and non‐refined oysters, were evaluated by Dutch consumers and the effect of information was also studied.

### Consumer panel

In all three studies consumers were recruited through advertisements in local newspapers and from a pool of consumers used in previous studies.^4^, ^5^, ^11^ The main selection criteria for the consumers was that they were consumers of oysters. The number of participating consumers varied between 56, 72, and 85 participants in studies 2, 3, and 1, respectively. Consumers were not paid for their participation and neither were they told the study's aim or experimental design. Table 1 shows the characteristics of the consumer panels.

**Table 1:** Sociodemographic characteristics of the consumer panels in different oyster evaluation studies. Study 1: Quality perception. Study 2: Purchase intent. Study 3: Consumer evaluation and impact information

	Study		
	1 (*n* = 85)	2 (*n* = 56)	3 (*n* = 72)
Gender (%)			
Female	35	23	42
Male	65	77	58
Age (years) (%)			
< 25	1	2	1
26–35	11	11	7
36–45	4	5	6
46–55	18	25	24
> 55	67	56	62
Weekly seafood consumption (%)			
Less than once	20	25	40
Once to twice	66	55	49
More than twice	14	20	12
Yearly frequency oyster consumption (%)			
Once a year	6	8	10
2–3	33	37	38
4–10	44	35	33
> 10	17	20	19
Highest educational level (%)^a^			
Low	7	4	7
Middle	69	65	65
High	24	31	28
Gross yearly income (%)			
≤ €32 999	35	27	31
€33 000 ‐ €49 999	51	45	50
≥ €50 000	14	27	19

a Low educational level: primary school and secondary school; middle educational level: intermediate vocational education and bachelor's degree or equivalents; high educational level: master's degree and doctor of philosophy degree or equivalents.

In all studies consumers were seated at random approximately 1 m apart in classrooms. They were instructed not to speak to each other and received a participation number for anonymity.

### Study 1: Quality perception

In two focus‐group sessions, scientists working in the field of oyster cultivation, oyster farmers, oyster traders, and chefs (*n* = 10) predefined oyster quality characteristics and sensorial properties. The agreed predefined oyster quality characteristics could be categorized into biometric (total weight, tissue weight, meat content, shell length, shell width, shell depth), phenotypical (shell shape, shell color, tissue color), sensorial (odor, taste, texture), and extrinsic (shelf life, country of origin, flavor profile, cultivation method, health claims, nutritional value, packaging) characteristics. The sensorial properties of oysters were further broken down into the following attributes: saltiness, pungency, sweetness, firmness, and creaminess. The predefined quality characteristics and sensorial properties have also been used in previous studies.^12^, ^13^

The questionnaires regarding the oyster quality characteristics and sensorial properties were distributed among the participating consumers (*n* = 85). The attribute definitions were explained to the consumers in order to ensure agreement about the terminology used. Consumers were asked to score the importance of the predefined oyster quality characteristics and sensorial properties on a seven‐point scale, anchored with ‘not at all important to me’ on the left‐hand side and ‘very important to me’ on the right‐hand side. The participants in the focus group sessions did not participate in these tests.

### Study 2: Purchase intentions

Consumers' purchase intentions in relation to country of origin, cultivation area, type of cultivation process and flavor profile were measured using questionnaires. A full factorial design was used to reveal how much these factors influenced willingness to buy and willingness to pay for oysters; each factor had two levels:

- Country of origin: domestic versus imported oyster, presented to the consumers as Dutch or Irish oysters.
- Cultivation area: natural versus cultivation waters, presented to the consumers as oysters cultivated in the Eastern Scheldt (a nature reserve area) or Lake Grevelingen (the most important Dutch oyster production area).
- Type of cultivation process: refinement versus no refinement, presented to the consumers as refined or non‐refined oysters.
- Flavor profile: including and excluding the attribute ‘sweet,’ presented to the consumers as saline, creamy, sweet or saline, creamy.

**Figure 1:**
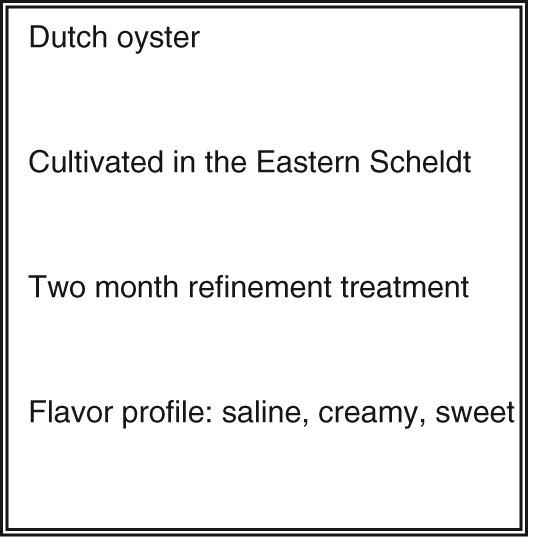
Example of oyster label.

The ‘sweet’ attribute was selected because perceived sweetness is known to change in refined oysters in comparison with non‐refined oysters.^3^, ^4^ These factors were combined in a virtual oyster label, which was presented in a questionnaire to the consumers (*n* = 56, see Fig. 1). Willingness to buy was assessed using a seven‐point scale ranging from ‘not at all’ on the left‐hand side to ‘surely’ on the right‐hand side of the scale. Willingness to pay was assessed using a seven‐point scale ranging from €0.50 to €2.00 per individual oyster. The range in price level is comparable with Dutch retail price levels. Low price levels (approximately €0.50 per oyster) have been recorded for domestic oysters in discount supermarkets whereas the high prices (approx. €2.00 per oyster) have been recorded for high‐quality imported French oysters in seafood specialty stores. Participating consumers were asked to mention reasons for the willingness to buy on the questionnaire forms.

### Study 3: Consumer evaluation and impact information

Actual products, being refined and non‐refined oysters, were evaluated by naïve consumers (*n* = 72) by rating the intensity of key attributes and acceptance. Alive refined and non‐refined Pacific cupped oysters were obtained from a shellfish company (Renart Boulon, Kamperland, Netherlands). Non‐refined oysters originated from the Lake Grevelingen cultivation area (Netherlands) and received no further treatment. Refined oysters also originated from the same cultivation area but were fed with a monoculture of microalgae for 1 month in land‐based pond systems. Oysters were obtained daily from the wet storage area of the shellfish company in order to ensure optimal quality. The oyster samples were stored refrigerated for a maximum of 4 h at 4–6 °C until preparation for the consumer test. Oysters were opened by hand‐shucking and the adductor muscles were cut with a knife on both sides. Prior to serving the prepared samples, the remaining internal liquid was drained from the oysters. In the tests, oysters were served as half‐shell products.

In total, six oysters were served in three rounds in the consumer evaluation test. Each round lasted approximately 15 min. Between rounds, consumers received a five‐minute break to take a sip of water or to eat a cracker to clean their palates. Samples assessed consisted of three refined and three non‐refined oysters. Two oysters (one refined and one non‐refined) were presented without any information on the cultivation process. The remaining four oysters were presented with information on the cultivation process (either refined or non‐refined oysters) by means of an accompanying label. Two of the oysters presented were correctly labeled (one refined and one non‐refined) and two were mislabeled (a refined oyster was labeled as being ‘non‐refined’ and vice versa). The presentation order of the samples was randomized.

The attributes in the consumer evaluation test were selected from studies regarding the sensory profile of oysters.^3^, ^5^, ^14^, ^15^, ^16^ The comprehensibility of the selected attributes was discussed in a preliminary session with ten consumers. The agreed attributes for the sensory profile test were: greenness, odor intensity, sea odor, mud odor, sweetness, saltiness, astringency, firmness, creaminess, and overall liking. The ten consumers from the preliminary session did not participate in the actual tests. The selected attributes were scored on a nine‐point scale ranging from ‘very low’ on the left‐hand side to ‘very high’ on the right‐hand side. The attribute definitions and scales were explained to the consumers in order to ensure agreement in the understanding of the terminology used and the intensity scores.

Using trained panels is the most common approach in sensory evaluations. However, in our study we wanted to compare the evaluations of the different oyster products by end users, i.e., naïve consumers. Previous studies^17^, ^18^ showed that the use of naïve consumer panels is a good alternative to using trained panels, even in complex products such as perfumes.

### Statistical data analysis

In study 1, the quality perception data from the questionnaires were ranked using Kruskal–Wallis tests. Differences between quality characteristics were evaluated using Wilcoxon signed rank tests.

The data from study 2 were analyzed using a one‐way analysis of variance (ANOVA) for the weighted averages based on factor scores to evaluate effects of country of origin, cultivation area, cultivation process, and flavor profile of oysters on consumers' willingness to buy and willingness to pay for the tested oyster labels. Reasons mentioned by consumers explaining their willingness to buy were categorized into themes (country of origin, cultivation area, type of cultivation process, and flavor profile).^19^ Reasons were considered positive when willingness to buy was scored above four, on a seven‐point scale. Likewise, reasons were considered negative when willingness to buy was scored below four. The categories mentioned are expressed as percentages in the results section.

In study 3, differences in the consumer evaluations of refined and non‐refined oysters were analyzed using one‐way ANOVA. Likewise, for both refined and non‐refined oysters, a one‐way ANOVA was used in order to evaluate the effect of providing information about the cultivation process on the consumer evaluations. As for the latter, post‐hoc Tukey analysis was applied when significant effects were found.

Results are reported as means ± standard deviation. Where *P* < 0.05, differences were deemed statistically significant.

## RESULTS

### Study 1: Quality perception

**Figure 2:**
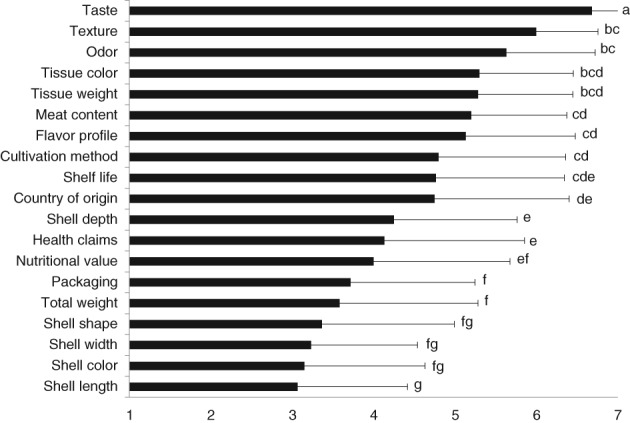
Mean (± standard deviation) importance of oyster quality characteristics according to Dutch consumers (*n* = 85). 1 stands for ‘not at all important’; 7 stands for ‘very important.’ Different superscripts indicate significant differences (*P* < 0.05).

Figure 2 shows that Dutch consumers regard sensory aspects such as taste (6.7 ± 0.5), texture (6.0 ± 0.8), and odor (5.6 ± 1.1) as important quality characteristics (χ^2^ = 418.524, *P* < 0.001).

Some of the phenotypical characteristics are also considered to be important as quality characteristics by the consumers. For instance, tissue color (5.3 ± 1.2) and meat content (5.2 ± 1.2) were considered important quality characteristics, whereas aspects like shell shape (3.4 ± 1.6) and shell color (3.2 ± 1.5) were considered to be less important.

Extrinsic characteristics such as cultivation method (4.8 ± 1.6), shelf life (4.8 ± 1.6), country of origin (4.8 ± 1.7), health claims (4.1 ± 1.7), and nutritional value (4.0 ± 1.7) were considered less important than the phenotypical characteristics. Biometric characteristics such as total weight (3.6 ± 1.7), shell width (3.4 ± 1.3), and shell length (3.1 ± 1.4) were considered the least important quality characteristics. Other biometric parameters like shell depth (4.3 ± 1.5) and tissue weight (5.3 ± 1.2) were considered to be more important as quality characteristics.

**Figure 3:**
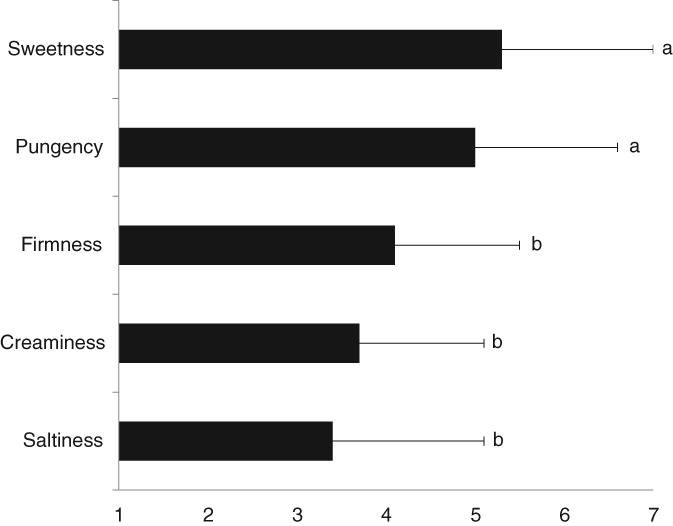
Mean (± standard deviation) importance of oyster sensorial properties according to Dutch consumers (*n* = 85). 1 stands for ‘not at all important’; 7 stands for ‘very important.’ Different superscripts indicate significant differences (*P* < 0.05).

From the five predefined sensorial properties, Dutch consumers rank sweetness and pungency as the most important (5.3 ± 1.7 and 5.0 ± 1.6, respectively) (Fig. 3). Firmness (4.1 ± 1.4),creaminess (3.7 ± 1.4) and saltiness (3.4 ± 1.7) were considered less important (χ^2^ = 57.875, *P* < 0.001).

### Study 2: Purchase intention

Table 2 shows the effects of different purchase intention factors on Dutch consumers' willingness to buy and willingness to pay for oysters. Significant effects were found for country of origin, cultivation area, and flavor profile. Consumers are more willing to buy domestic oysters in comparison with imported oysters (F = 25 860, *P* < 0.001). The average price that consumers are willing to pay for domestic oysters is higher in comparison to the average price for imported oysters (F = 9.382, *P* = 0.002). Whether oysters originated from natural waters or specific cultivation waters did not affect consumers' willingness to buy oysters significantly (F = 1.751, *P* = 0.187). However, consumers were willing to pay more for oysters from natural waters than for oysters from cultivation areas (F = 4.125, *P* = 0.043).

**Table 2:** Effect of factors on oyster purchase intent (*n* = 56): country of origin, cultivation area, cultivation process and flavor profile on consumer's willningness to buy (mean ± standard deviation), based on a seven‐point scale from 1 = not at all to 7 = surely) and willingness to pay (€ per oyster). Different superscripts indicate significant differences (*P* < 0.05).

	Country of origin		Cultivation area	
	Native	Non‐native	Natural waters	Cultivation waters
Willingness to buy	5,3^b^ ± 1,6	4,5^a^ ± 1,8	5,4 ± 1,6	5,1 ± 1,8
Willingness to pay	1,20^b^ ± 0,64	1,07^a^ ± 0,64	1,23^b^ ± 0,63	1,10^a^ ± 0,65
	Cultivation process		Flavor profile	
	Refined	Non‐refined	Incl. sweet	Excl. sweet
Willingness to buy	5,1 ± 1,8	4,9 ± 1,7	5,3^b^ ± 1,7	4,5^a^ ± 1,8
Willingness to pay	1,14 ± 0,65	1,15 ± 0,64	1,18^b^ ± 0,64	1,08^a^ ± 0,64

Consumers were more willing to buy (F = 21.092, *P* < 0.001) and more willing to pay (F = 5.755, *P* = 0.017) for oysters that included the description ‘sweet’ in the flavor profile when compared to oysters accompanied with a flavor profile excluding the description ‘sweet.’ No effect of treatment was found on the consumers' willingness to buy (F = 1.292, *P* = 0.256) and willingness to pay (F = 0.088, *P* = 0.767) for oysters.

The reasons consumers reported as influential for their willingness to buy could be grouped into the following categories: country of origin, cultivation area, cultivation process and flavor profile. The flavor profile (51% of all reasons mentioned by consumers) and country of origin (27%) seemed to be the most important reasons for willingness to buy. Cultivation area (17%) and cultivation process (4%) scored lower. All categories were scored as reasons for low as well as high willingness to buy oysters. Low willingness to buy is mostly due to the country of origin (44%) followed by the flavor profile (34%), cultivation area (18%), and cultivation process (4%). High willingness to buy is mostly due to the flavor profile (69%), followed by cultivation area (17%), cultivation process (15%), and country of origin (10%).

### Study 3: Consumer evaluation and impact of information

**Figure 4:**
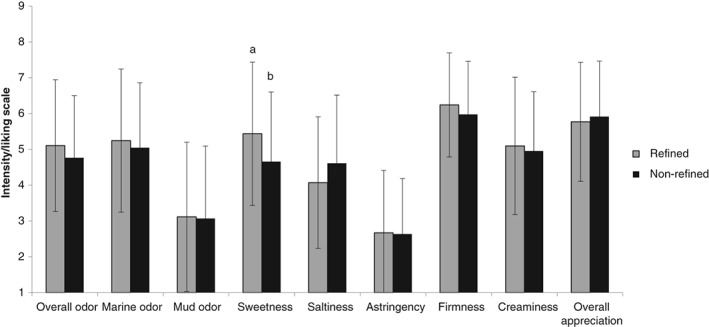
Mean (± standard deviation) consumer scores of sensorial properties (*n* = 72) of refined and non‐refined Pacific cupped oyster (Crassostrea gigas). 1 stands for ‘very low’; 7 stands for ‘very high.’ Different superscripts indicate significant differences (*P* < 0.05).

Dutch consumers' evaluation of Pacific cupped oysters showed significant differences between refined and non‐refined oysters for the ‘sweetness’ attribute (Fig. 4). Refined oysters were perceived as being sweeter than non‐refined oysters (5.4 ± 2.0 and 4.7 ± 1.9, F = 6.582, p = 0.011 respectively). The refinement procedure did not lead to an increased overall appreciation by the consumers (F = 0.336, *P* = 0.563).

**Figure 5:**
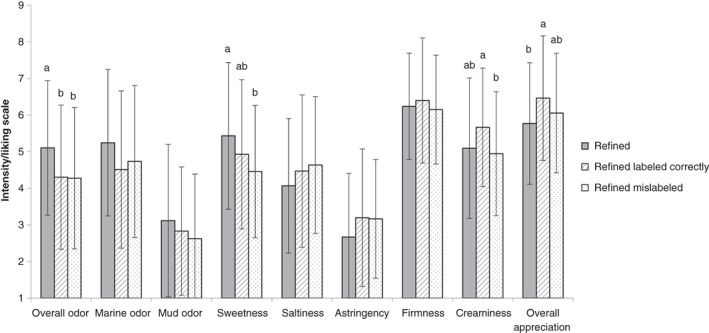
Mean (± standard deviation) consumer scores of sensorial properties (*n* = 72) of refined Pacific cupped oyster (Crassostrea gigas) without information on the cultivation process and with correctly labeled or mislabeled information on the cultivation process. 1 stands for ‘very low’; 7 stands for ‘very high.’ Different superscripts indicate significant differences (*P* < 0.05).

**Figure 6:**
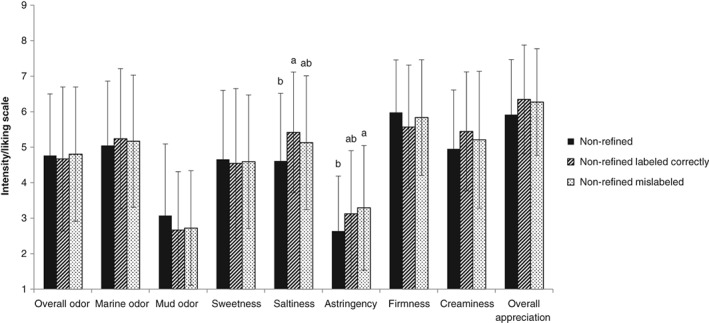
Mean (± standard deviation) consumer ratings of sensorial properties (*n* = 72) of non‐refined Pacific cupped oyster (Crassostrea gigas) without information on the cultivation process and with correctly labeled or mislabeled information of the cultivation process. 1 stands for ‘very low’; 7 stands for ‘very high.’ Different superscripts indicate significant differences (*P* < 0.05).

Consumers' evaluations changed when information on the cultivation process was provided (Figs 5 and 6). In the case of refined oysters, overall odor intensity was perceived as being less intense when information on the cultivation process was given. The score varied from 5.1 ± 1.8 without information to 4.3 ± 2.0 for the correctly labeled refined oysters and 4.3 ± 1.9 for the mislabeled refined oysters (F = 4.864, *P* = 0.009). Furthermore, a significant difference (F = 4.890, *P* = 0.008) was found between the perceived sweetness of refined oysters without cultivation information (5.4 ± 2.0) and refined oysters, which were mislabeled as non‐refined (4.5 ± 1.8). For the ‘creaminess’ attribute, a significant difference (F = 3.417, *P* = 0.035) was found for refined oysters, which were either correctly labeled (5.7 ± 1.6) or mislabeled (4.9 ± 1.7).

The consumers' overall appreciation of the oysters increased by providing them with information on the refinement cultivation process. A significant difference (F = 3.265, *P* = 0.040) was found between refined oysters without information on the cultivation process (5.8 ± 1.7) and refined oysters that were correctly labeled as being refined oysters (6.5 ± 1.7).

In the case of non‐refined oysters, perceived saltiness and astringency increased, albeit not in all cases significantly. When information on the cultivation process (no refinement) was provided to the consumers, significant differences (F = 3.899, *P* = 0.022) were found between perceived saltiness of non‐refined oysters without information about the cultivation process (4.6 ± 1.9) and non‐refined oysters, which were correctly labeled as being non‐refined (5.4 ± 1.7). Likewise, the difference between the perceived astringency of non‐refined oysters without information on the cultivation process (2.6 ± 1.5), and non‐refined oysters, which were mislabeled as being refined oysters (3.3 ± 1.8), was also significant (F = 3.253, *P* = 0.040).

## DISCUSSION

Our aim was to gain insight into the importance of oyster quality parameters, drivers for oyster consumption of Dutch consumers and their acceptance for new oyster products such as refined oysters. Results show that consumers regard taste, texture, odor, tissue color, and meat content as the most important quality characteristics for oysters. Biometric parameters such as total weight, shell shape, width, and length were considered the least important characteristics. The importance of sensory aspects such as taste as a quality characteristic is not surprising. Several authors^13^, ^20^, ^21^ found that taste, texture, and odor are the main drivers for the consumption of oysters by US consumers. Texture was mentioned as one of the most important drivers for not consuming oysters.^20^ Furthermore it was shown that French consumers do not pay attention to the shell shape of the oysters, whereas a high meat content is preferred by the majority of the consumers.^13^ The importance of meat content and the appearance of the oyster as quality characteristics were also emphasized by Ruello.^22^

Effects of purchase intention factors (land of origin, cultivation area, and flavor profile) on consumers' willingness to buy and willingness to pay for oysters was clearly shown. As for country of origin, consumers showed a preference for domestic (Dutch) oysters in comparison to imported (Irish) oysters. Preference for domestic oysters was also seen in another questionnaire.^23^ Loureiro and Umberger^24^ suggested that consumers associate land of origin with aspects such as food safety and freshness, this explaining a preference for domestic products.

Results from the questionnaires in study 2 show that consumers prefer oysters cultivated in natural waters over oysters from known cultivation waters, although no realistic samples were evaluated. Natural waters are likely associated with concepts such as ‘nature,’ ‘pristine,’ or ‘clean.’ Siegrist^8^ suggested that concepts such as ‘nature’ and ‘naturalness’ related to food are positively valued by consumers. This positive association may explain the consumers' preferences in our study. In contrast, French consumers showed preference for oysters from renowned cultivation areas in a national questionnaire.^13^ This preference is most likely due to the greater familiarity of French consumers with the cultivation areas. In France, the cultivation area of the oysters is used as a distinctive marketing tool.

In our second study, flavor profiles including the attribute ‘sweet’ were given a higher score by consumers than flavor profiles without it. Furthermore, results show that Dutch consumers valued sweetness as the most important flavor characteristic, while saltiness was considered the least important. This suggests a preference of Dutch consumers for the flavor profile of refined oysters. Without actually tasting the refined or non‐refined oysters, consumers' willingness to buy or willingness to pay for refined and non‐refined oysters showed no significant differences. As oyster refinement is a new cultivation method that is not well known in the Netherlands, this might have affected the consumers' willingness to buy and willingness to pay for them. Trust of new food technology and consumers' lack of knowledge about it are known to influence consumer perceptions.^25^, ^26^, ^27^, ^28^, ^29^ Verbeke^26^ showed that new food technologies might evoke expressions of ‘disgust,’ ‘unnaturalness,’ or ‘fear’ and might lead to negative evaluations. Lee^27^ showed that information on the food technology applied can lead to increased consumer trust towards the technology used. Our results show no lack of consumer trust towards refined oysters but it has to be remarked that no actual products were bought during this study. In reality both willingness to buy and willingness to pay might be different; the results should therefore be used with caution.

When consumers evaluated both the refined and non‐refined oysters, they perceived refined oysters as being sweeter than non‐refined oysters. This evaluation was done without providing information on the cultivation process. Providing information on the cultivation process of the oysters (being either refined or non‐refined) affected evaluation by consumers. Overall appreciation of refined oysters increased when consumers were aware of the refinement. Furthermore, odor intensity and marine odor perception decreased for refined oysters while creaminess perception increased. For non‐refined oysters, information about the cultivation process led to an increase in saltiness perception by the consumers. Providing false information on the cultivation process (refined being labeled as non‐refined and vice versa) only lowered the perceived creaminess of the refined oysters. Caporale and Monteleone^30^ suggested that information on food processing may influence how the taste of a product is evaluated. Moreover, information, or the lack thereof, has been shown to influence the willingness to buy and expected liking.^27^, ^31^, ^32^, ^33^, ^34^ Providing information could increase willingness to buy and expected liking of the product in question. In the case of mislabeling, it has been shown that consumer overall liking is significantly influenced by providing false information in mislabeled red wines.^35^ Prior to actual tasting, expectations did not show any effects of mislabeling as the expectation of the falsely labeled wines was equal to the correctly labeled wines.

Some of the limitations of this study include the lack of price aspect as a driver for consumer purchase intent. It has been shown that the price aspect is the strongest driver for consumer intent to purchase seafood in general^36^, ^37^, ^38^ and oysters in particular.^13^, ^23^ The aspect of price is not taken into account as an oyster quality characteristic in our study. Like most consumer studies, we did not include a price variable as it might have reduced the variation in the rest of the attributes in the consumer evaluations. Furthermore, no real money and products were involved as willingness to pay in our study was assessed using questionnaire data. The drawback of using questionnaire data is that it might lead to higher measured willingness to pay in comparison with real‐life settings involving the actual purchase of the products.^39^, ^40^ In our study we were not interested in defining a realistic price for the oysters tested but in differences in willingness to buy and willingness to pay between the products.

Our study gives new insight into the importance of quality and flavor characteristics for oyster consumers. These consumer insights can be used in the development of new oyster products. Furthermore, the results of our study show that Dutch consumers might prefer and buy refined oysters as the flavor profile of refined oysters is more in agreement with the preference of Dutch consumers. No apparent negative attitude from the consumers towards the refinement process was observed in our studies. Providing the consumers with information on the production process leads to significant changes in the consumer evaluations of the oysters. Moreover, by showing the importance of some intrinsic and extrinsic product characteristics to oyster consumers, this study also provides an insight into some psychological factors affecting their choices and evaluations.
